# 3D patient-specific modeling and structural finite element analysis of atherosclerotic carotid artery based on computed tomography angiography

**DOI:** 10.1038/s41598-023-46949-5

**Published:** 2023-11-14

**Authors:** Nicoletta Curcio, Antonio Rosato, Daniela Mazzaccaro, Giovanni Nano, Michele Conti, Giulia Matrone

**Affiliations:** 1 Department of Electrical, Computer and Biomedical Engineering, University of Pavia, Pavia, Italy; 2 3D and Computer Simulation Laboratory, IRCCS Policlinico San Donato, San Donato Milanese, Italy; 3 Operative Unit of Vascular Surgery, IRCCS Policlinico San Donato, San Donato Milanese, Italy; 4 Department of Biomedical Sciences for Health, University of Milan, Milan, Italy; 5 Department of Civil Engineering and Architecture, University of Pavia, Pavia, Italy

**Keywords:** Atherosclerosis, Carotid artery disease, Biomedical engineering

## Abstract

The assessment of carotid plaque vulnerability is a relevant clinical information that can help prevent adverse cerebrovascular events. To this aim, in this study, we propose a patient-specific computational workflow to quantify the stress distribution in an atherosclerotic carotid artery, by means of geometric modeling and structural simulation of the plaque and vessel wall. Ten patients were involved in our study. Starting with segmentation of the lumen, calcific and lipid plaque components from computed tomography angiography images, the fibrous component and the vessel wall were semi-automatically reconstructed with an ad-hoc procedure. Finite element analyses were performed using local pressure values derived from ultrasound imaging. Simulation outputs were analyzed to assess how mechanical factors influence the stresses within the atherosclerotic wall. The developed reconstruction method was first evaluated by comparing the results obtained using the automatically generated fibrous component model and the one derived from image segmentation. The high-stress regions in the carotid artery wall around plaques suggest areas of possible rupture. In mostly lipidic and heterogeneous plaques, the highest stresses are localized at the interface between the lipidic components and the lumen, in the fibrous cap.

## Introduction

Stroke is the main cause of long-term disability and the third leading cause of death^1^. Large-artery atherosclerosis, specifically carotid artery (CA) stenosis, is a major cause of all ischemic strokes^2^. Atherosclerosis is a systemic disease that can affect vessel branches and areas that are exposed to complex blood flow^3^. Carotid arteries represent a preferential site for the development of atherosclerotic plaques. The progression of these pathologic lesions depends on some factors, and can be precipitated by the influence of mechanical forces exerted on vessel walls. This pathological condition is expressed by the remodeling and subsequent reduction of the arterial lumen, due to the accumulation of inflammatory cells, lipids, extracellular matrix, and other materials in the inner layer of artery walls, which lead to the formation of atherosclerotic plaque.

Since atherosclerotic lesions develop over time, they can be found in several stages, going from an initial, asymptomatic, and non-stenotic stage to an advanced stage that can lead to several acute cardiovascular events^4^. Besides the degree of stenosis, from a morphologic point of view, atherosclerotic plaques can be classified as stable or vulnerable. A stable plaque is generally calcified and/or can have a small lipid core which shows a thick fibrous cap (FC). On the other hand, unstable plaques can easily rupture, causing severe cardiovascular events, such as stroke^5^. At a macroscopic level, the latter type of plaques may have a huge lipid core (LC), a thin FC that covers the LC, and may have features of intraplaque hemorrhage (IPH), neovascularization, necrosis, small calcification, and surface ulceration^6^. In particular, the FC represents the portion of the plaque that faces the vascular lumen and consists of vascular smooth muscle cells embedded in a matrix containing collagen fibers. The FC maintains the plaque's integrity and could rupture, causing the cholesterol accumulated in the LC to leak out and the exposition of the extracellular matrix to blood^5^, with consequent activation of the extrinsic coagulation pathway and triggering of a local pro-thrombotic status. Atherosclerotic plaque rupture is an event that occurs when the vessel wall mechanical loading exceeds the tissue strength. The plaque morphology and composition influence the stress levels and, consequently, the mechanical stability of the plaque itself^7^. Conversely, mechanical loading regulates the cellular and molecular composition of plaques^3^. Sometimes, albeit not frequently, cardiovascular complications that derive from the rupture of a vulnerable plaque could be asymptomatic^6^. Some studies also highlighted that unstable plaques can cause severe cardiovascular events regardless of the degree of stenosis^8,9^.

Identifying patients with plaques that are likely to be vulnerable represents an important step to prevent acute and adverse cardiovascular events. Computational studies provide the three-dimensional (3D) stress/strain distributions in patient-specific diseased arteries of different types and morphology, so they could have the potential to estimate the risk of plaque rupture. Several groups have considered different strategies for plaque vulnerability assessment, by integrating clinical imaging techniques with computational analyses. Teng et al.^10^ conducted an in vivo study investigating the association between the vulnerable site (as confirmed by histological data of plaque rupture) and the maximum wall stresses. The results of plaque wall stress analysis at the critical sites matched the histology-confirmed rupture sites with an 83% agreement. Most of the studies considered cases of disrupted plaque or with LC portions. Gao et al.^11^ analysed the effects of LC volume and FC thickness on stress distribution in carotid plaques. Wall stress was higher in the luminal wall and lower in the outer wall, with the lowest stress in the lipid region. In the FC region, local stress concentrations were higher where the cap thickness decreased. Other works suggested that LC size and FC thickness are the structural features connected to plaque vulnerability^12,13^.

Some studies evaluated the role of calcific content in carotid plaques too. Calcification is also commonly found in advanced atherosclerosis plaques. Teng et al.^14^ evaluated how the presence of juxtaluminal calcium affects the critical mechanical conditions within carotid plaques. Calcific plaques showed high-stress concentration, but if a layer of thin FC was used to artificially cover the calcium, the high-stress local concentration disappeared. However, the impact of calcific content on plaque vulnerability is still unclear. Indeed, Benitez et al.^15^ investigated the effect of different calcification patterns and shapes. Their paper also underlined that it is unknown if calcifications provide stability to the plaque or may increase the risk of plaque rupture.

In the majority of studies mentioned above, the identification of the full plaque and the vessel wall reconstruction are considered a challenging task. Indeed, it is difficult to detect and distinguish the different plaque components based on in vivo clinical imaging techniques.

The “gold standard” for determining plaque geometry and composition is histology. Histology provides excellent resolution and clear delineation of tissue composition, but it is known that reconstruction of the vessel wall and plaque models based on histological images can cause geometry artifacts^16^.

Computed tomography angiography (CTA) is generally used in the clinical routine to investigate the stenosis degree of atherosclerotic vessels and to evaluate the plaque morphology and components^4,17^.

The main advantages of CTA are the high spatial and contrast resolution, and short times of acquisition^18,19^. In the vascular field, however, CTA is limited to the visualization of the vessel lumen and does not provide information regarding the structure of the vessel wall^19^. Magnetic Resonance Imaging (MRI) can well characterize carotid plaque structure and assess its composition too, with generally higher soft tissue discrimination capabilities than CT imaging; however, it is an expensive examination with limited availability and lengthy examination duration, thus not suitable for screening purposes^20^. Besides, ultrasound (US) is often used to perform a first evaluation of CA stenosis, and also to measure intima-media thickness (IMT), which is a known marker of systemic atherosclerosis^5^. In particular, Doppler ultrasound can help characterize the plaques, and identify those at high risk of embolization. US elastography has been used for plaque characterization too^20^. However, a major limitation is that US acquisitions are user dependent, and US-based atherosclerotic wall geometry reconstruction is difficult, due e.g. to the poor image quality resulting from artifacts, like shadowing caused by calcified plaques, which makes segmentation in the cross-sectional plane challenging.

Therefore, considering that CTA and MRI are the most accurate non-invasive methods in evaluating morphological characteristics of carotid plaques, computational analyses are mainly based on these imaging methods. Indeed, many works in the literature have derived the atherosclerotic vessel geometry for Finite Element Analysis (FEA) from MRI^11,12,14,15,21^ and CTA images^13,22–26^. Some studies^27–29^ also demonstrated the feasibility of 3D US-based segmentation and reconstruction of the CA wall and lumen affected by atherosclerosis disease, by modeling the plaque as the region enclosed between the wall and stenotic lumen. Arsenescu et al.^30^ performed segmentation of the plaque distinguishing the soft content from the calcified one. These 3D US-based models can also be used for further computational simulations; nevertheless, to the best of our knowledge, a few studies performed blood flow simulations only^31,32^. Also Intra Vascular US (IVUS), combined with angiography data, has been used to reconstruct atherosclerotic vessels and to evaluate structural stress using the finite element (FE) approach^33–35^.

In this context, the aim of our work is to develop a workflow to quantify the stress distribution of an atherosclerotic CA by means of 3D patient-specific vessel modelling and structural simulations. To do so, firstly we propose a methodology to reconstruct the patients’ atherosclerotic carotid walls, starting from CTA images, with an ad-hoc developed semi-automatic procedure. Particularly, thanks to CTA-based image analysis, we identify and reconstruct the calcific and lipid components geometry. In addition, the proposed method also allows to automatically generate the model of the plaque fibrous component, which usually cannot be easily segmented in CTA images and can play a relevant role in the evaluation of plaque vulnerability, especially where a lipid core surrounded by the FC is present. The modelling of the fibrous component represents a higher degree of detail compared to the other computational studies mentioned before. It allows to model more accurately the atherosclerotic vessel by considering different mechanical properties for each plaque component and arterial walls, as well as to make a more accurate selection of the volume of interest for stress distribution analysis. Indeed, in this way, we aim to overcome current limitations of other studies regarding CA model reconstruction based on CTA images, which used for example manual segmentation to reconstruct plaque components and wall structure geometries^11,12,14^, or performed a semi-automatic segmentation for lipid and calcific plaque geometries only^13^, or modelled idealized plaque geometries^23^ while the fibrous and healthy wall tissues surrounding the plaques were again reconstructed manually.

Secondly, in this present study, patient-specific local pressure data obtained from ultrasound (US) imaging modality are used to set biomechanical vessel loads in our simulations.

The patient-specific geometric modelling of atherosclerotic CA here described, together with the assessment of wall stress distribution around plaques through 3D FEA, can provide valuable information to identify vulnerable plaques.

## Results

**Figure 1:**
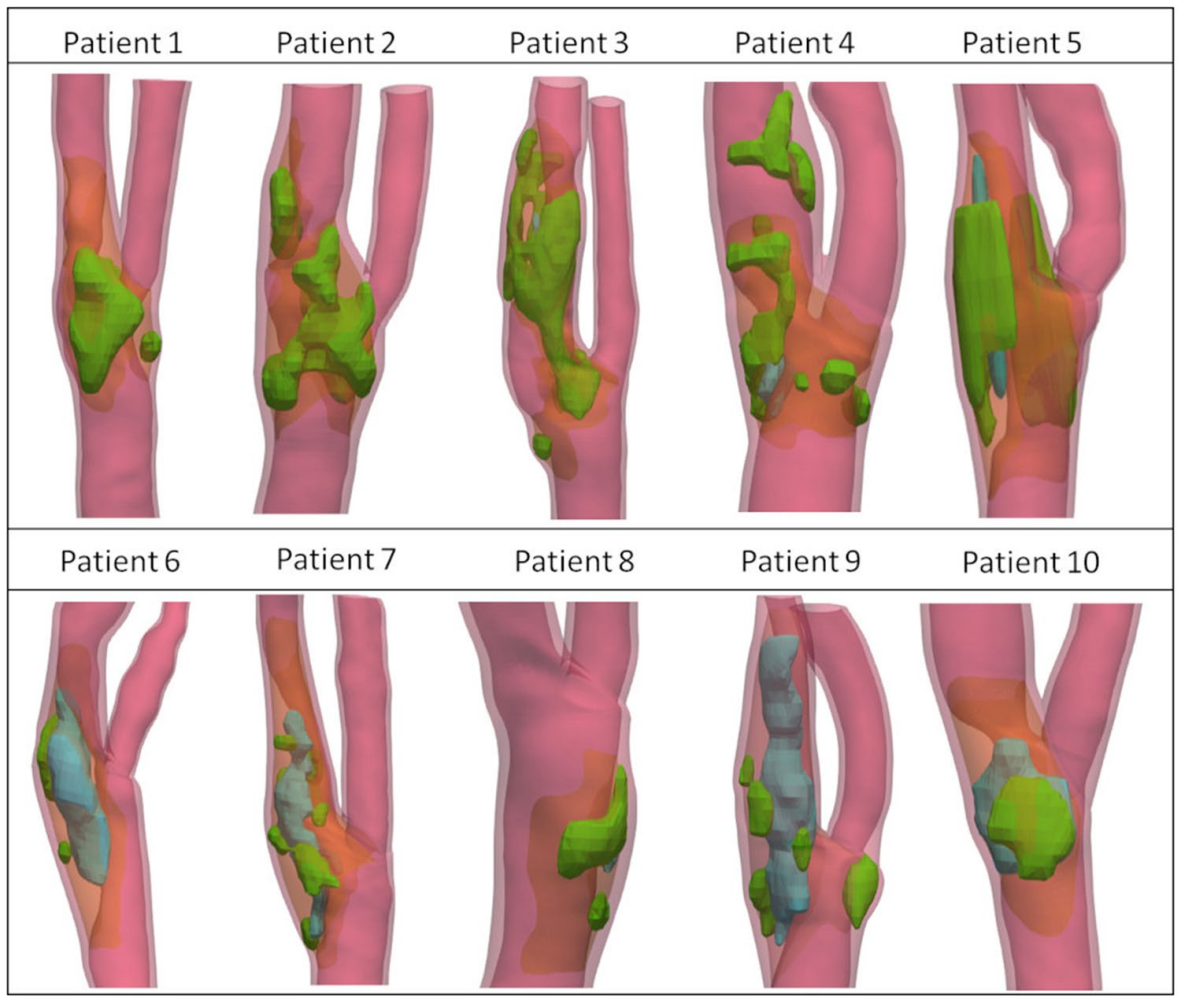
Final patient-specific atherosclerotic CA wall geometries of the ten patients, obtained from the segmentation of in vivo images, with all macro-components that can be present in an atheromatous plaque, i.e. calcific (green), lipid (blue) and fibrous (orange) components.

In Fig. 1, the ten final patient-specific atherosclerotic wall geometries, obtained from in vivo images, are represented with all the main components that may be present in an atheromatous plaque.

### Evaluation of the proposed model

**Figure 2:**
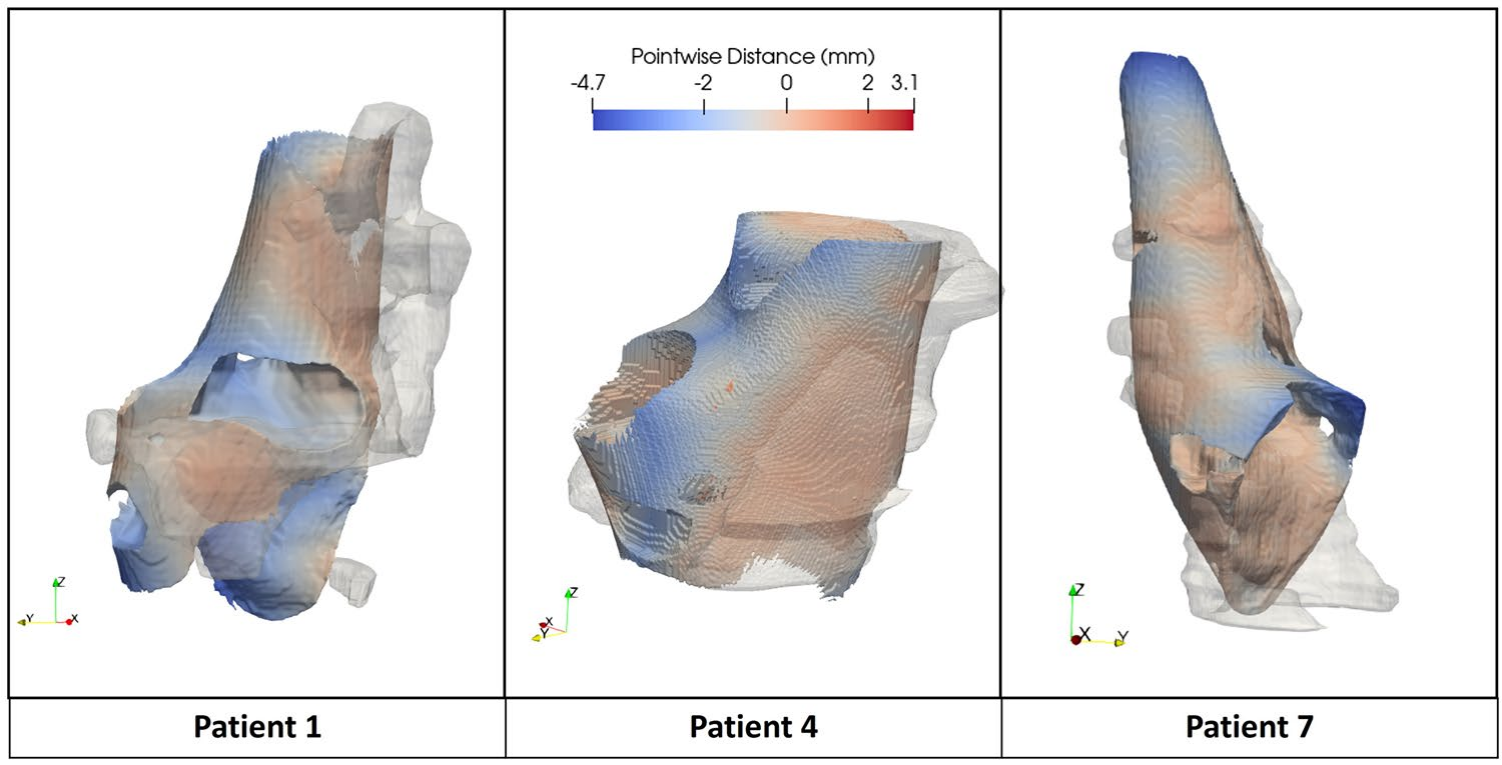
Pointwise distances between the reference (in transparency) and proposed models of the fibrous plaque component for patients 1, 4 and 7.

The first step of our model evaluation involved an analysis of mutual distances between the proposed reconstructed geometry and the manually segmented geometry of the plaque fibrous components (see Methods section). Results are described here and in Fig. 2 for patients 1–4–7 only, while for the seven remaining patients they are provided in the Supplementary Material (Section 2). Figure 2 represents the fibrous tissue models of patients 1–4–7, including both the proposed computer aided design (CAD) model and the segmented one. The color scale shows the mutual distance between each model, with positive values (in red) indicating that the fibrous model obtained from the manual segmentation, considered as a reference for the comparison, is larger than the CAD model (i.e., outside its surface). The average signed distance between the STL models of the fibrous component obtained from manual segmentation and from the proposed reconstruction procedure was equal to −1.356 ± 1.079 mm (mean ± standard deviation), −0.509 ± 0.746 mm and −0.399 ± 0.786 mm for patients 1, 4 and 7, respectively. Considering all the 10 patients instead, the distance was −0.169 ± 0.622 mm (mean ± standard deviation) on average, and ranged from −1.544 mm to 0.547 mm (5th and 95th percentile, respectively).

**Figure 3:**
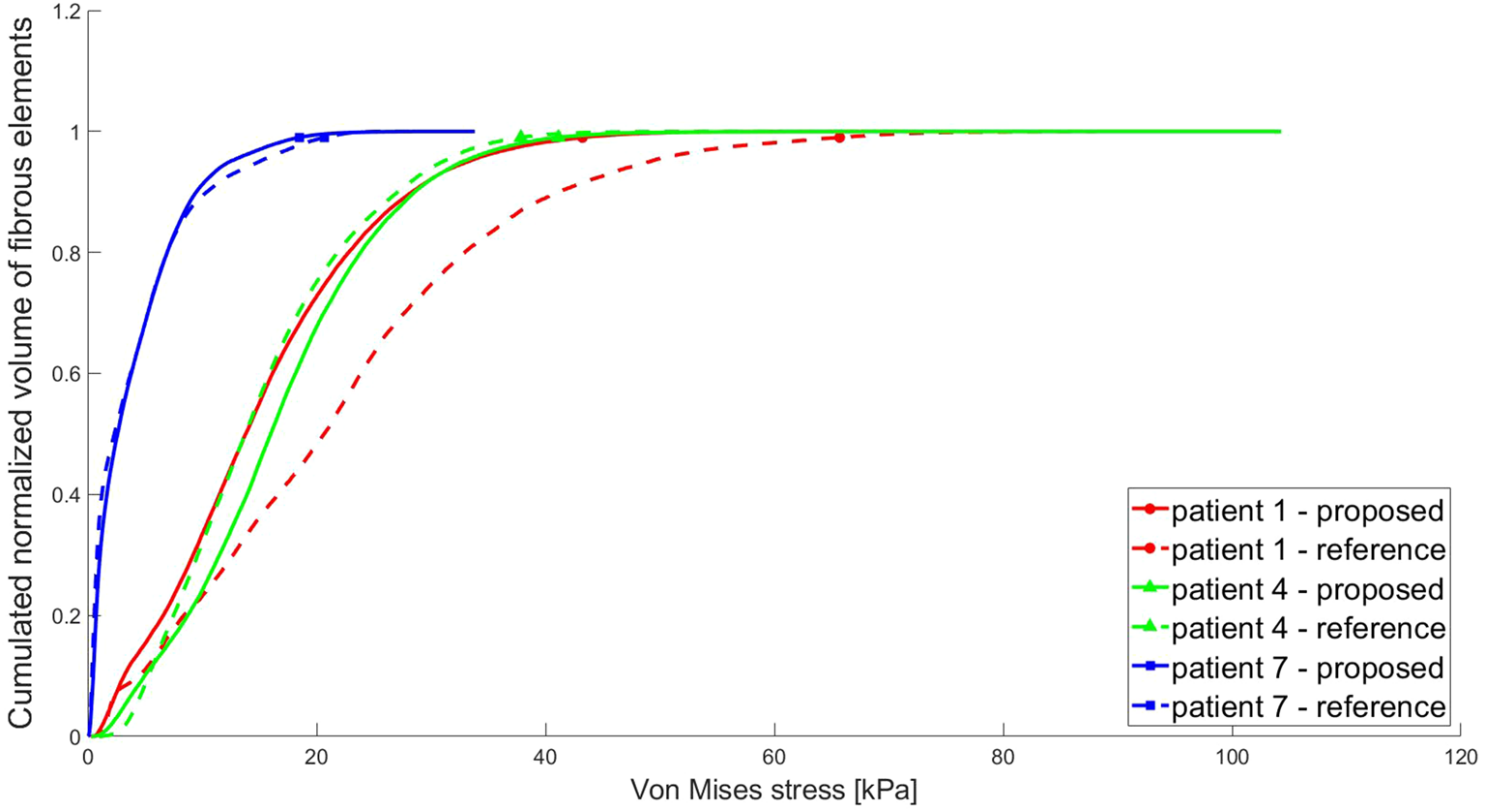
VM stresses as a function of the cumulative normalized volume of the fibrous plaque elements. For each case (i.e. patients 1, 4, 7, identified by the different colors) the volume-stress plots are shown with different lines that represent simulations results using either the proposed model (solid curves) or the reference one (dotted curves). The markers highlight VM_99_ stress values for each simulation/patient.

**Figure 4:**
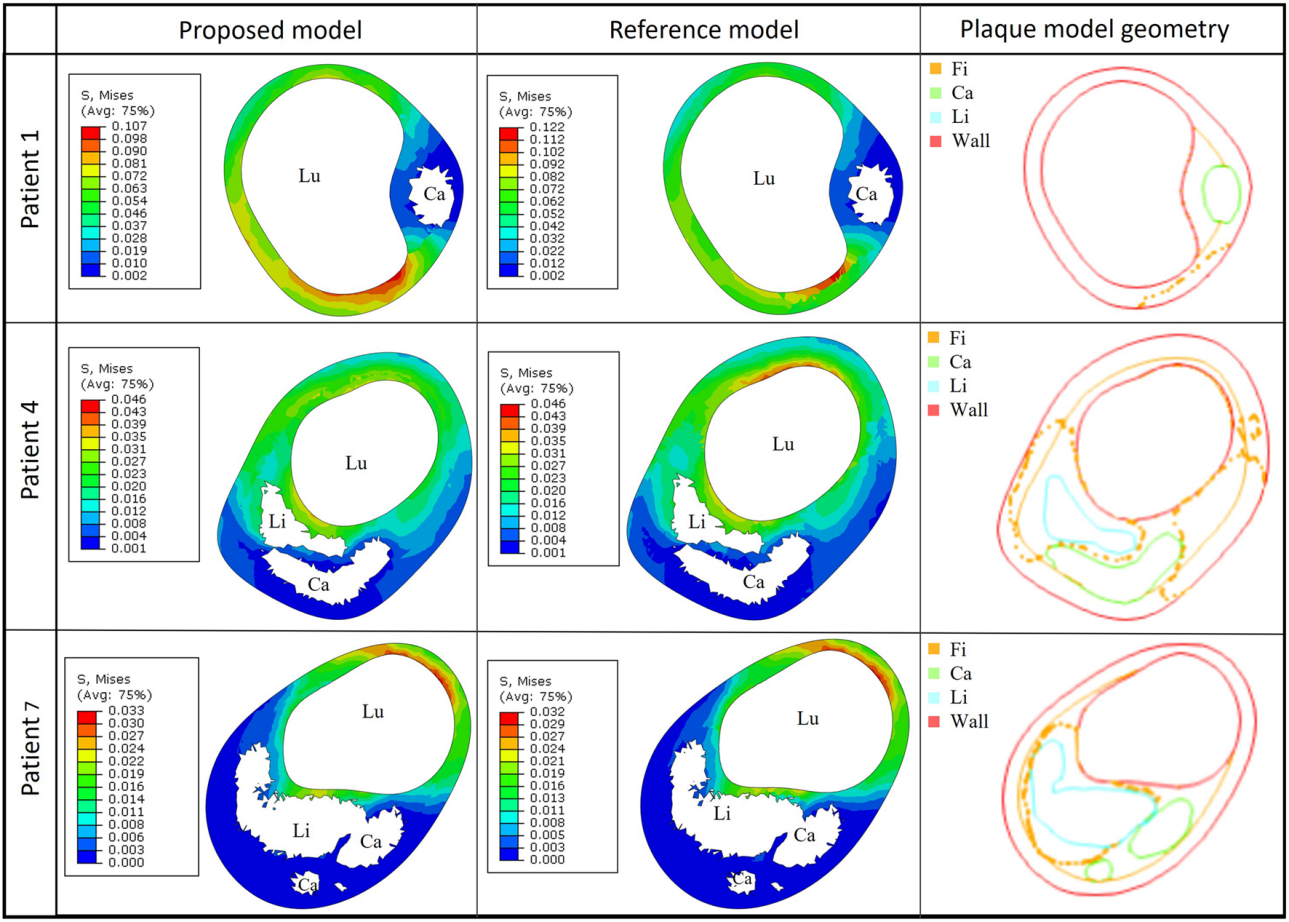
Results of the comparative analysis for three selected cases (patient 1, 4, 7). The cross-sectional contour plots of VM stress distributions (in MPa) in the CA healthy wall and fibrous plaque, obtained from simulations of the proposed model and reference model, are shown in the figure (left and middle panels), together with the corresponding 3D model sections derived from the STL files (on the right). The lumen is denoted as Lu, while the lipid, calcific and fibrous subsets are denoted as Li, Ca, and Fi, respectively. In the plots on the right, the orange solid and dotted curves for the fibrous component refer to the proposed and reference model, respectively.

To compare the simulation results obtained with the proposed model (obtained by applying the reconstruction procedure presented in the Methods) and the reference model (derived from the surgeon’s manual segmentation of all plaque components) in three selected cases (patients 1–4–7), we then plotted the increasing von Mises (VM) stress values as a function of the cumulative normalized volume of the fibrous subset, as shown in Fig. 3. In Table 1, we reported the VM_99_ stress values for each simulation, together with the volume of each fibrous component modelled, and the percentage differences obtained for these values using the proposed and reference models. To further analyze the obtained stress distributions within the CA healthy wall and fibrous subset, for each patient, we extracted the z-coordinate of the centroid of the element characterized by a VM stress value equal to VM_99_ in the simulation output generated from the reference model. These coordinates were then used to select the same cross-sectional slices in both simulation outputs (since both the models are equal except for the fibrous component, they share the same spatial reference system). This ensured that the slices displayed included the specific element characterized by the VM_99_ stress value. In Fig. 4, the contour plots of the obtained VM distributions are compared, together with the corresponding geometric model cross-sections.

**Table 1:** VM_99_ and fibrous component volume comparative analysis for patients 1, 4, 7.

Patient	Proposed model		Reference model		Percentage difference	
	VM_99_ (kPa)	Fibrous component volume (mm^3^)	VM_99_ (kPa)	Fibrous component volume (mm^3^)	VM_99_ (%)	Fibrous component volume (%)
1	43.23	174.54	65.71	92.65	− 34.22	88.39
4	41.14	236.72	37.85	199.82	8.69	18.46
7	18.5	618.43	20.09	449.89	− 7.91	37.46

Figure 3 and Table 1 demonstrate that the stress-volume curves and VM_99_ stress values obtained with the proposed and reference model are similar for patients 4 and 7. The curves and VM_99_ values of patient 1 instead show some more significant differences. This is due to the different shape of the fibrous component manually segmented by the surgeon as compared to the automatically reconstructed one, which also features a different volume, as can be observed by looking at Fig. 4 (panels on the right) and Table 1, as well as from the mutual distance analysis results which show higher differences in this case. Nevertheless, the simulated VM stress distributions presented in Fig. 4 look qualitatively similar in both models for all patients, as well as the location of the highest stress concentration.

In order to understand if such similarity was due to the reduced difference between the Young’s moduli of the carotid wall (550 kPa) and plaque fibrous component (400 kPa), a sensitivity analysis was performed by varying this latter parameter by ± 50% at most. Results shown in the Supplementary Material (Section 3) for the three selected patients however reveal that, even when the Young’s moduli become quite different (i.e. 550 kPa vs. 200 kPa for the carotid wall and fibrous plaque, respectively), the reference and proposed models achieve again similar stress distributions.

### Plaque stress distribution

In order to evaluate the simulation outputs, a quantitative analysis of cumulative volume vs. stress curves was carried out for all patients, as previously described for patients 1, 4 and 7. In this case, the curves were not used for comparative purposes, but only to extract the VM_99_ values within the fibrous plaque subset for each case, which are reported in Table 2. In the table, the composition of the plaque is also described in terms of the relative percentage volumes of each component (calcific/lipid/fibrous).

**Table 2:** Summary of simulation results for all the analyzed cases.

Patient	Calcific component of the plaque (%)*	Lipid component of the plaque (%)*	Fibrous component of the plaque (%)*	VM_99_ stress (kPa)	VM_99_ location	FC thickness (mm)**
1	42.17	0	57.83	43.23	Calcific plaque shoulder	–
2	68.15	0	31.84	31.54	Calcific plaque shoulder	–
3	48.14	0.50	51.36	30.60	Calcific plaque shoulder	–
4	25.94	6.20	67.82	41.14	Bifurcation	–
5	48.15	12.03	39.82	28.30	Fibrous cap	0.150
6	8.69	48.65	42.66	52.61	Fibrous cap	0.475
7	8.76	20.94	70.30	18.50	Bifurcation	–
8	26.34	5.34	68.32	28.71	Fibrous cap	1.076
9	11.36	29.58	59.05	44.41	Fibrous cap	0.496
10	9.84	30.21	59.95	19.13	Bifurcation	–

*The values reported for the plaque components represent % volumes with respect to the plaque total volume.

**The thickness of the fibrous region was measured over the cross-sectional plane where the VM_99_ stress was detected.

**Figure 5:**
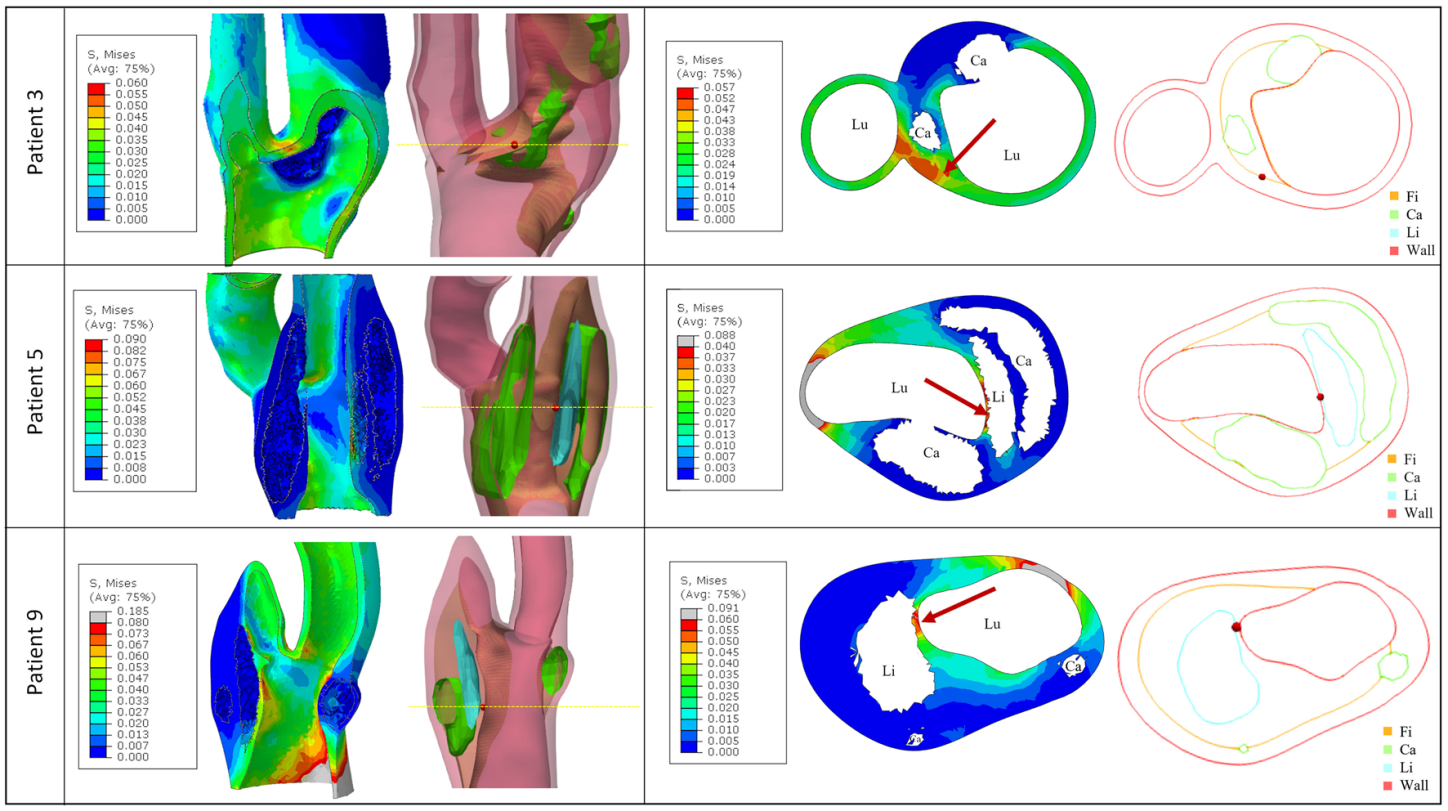
Contour plots of VM stress distributions (in MPa) in three example cases (patients 3, 5, 9), with the corresponding models of the vessel wall (red), and of the fibrous (orange), calcific (green) and lipid (light-blue) plaque components. The 3D views are provided on the left, while the cross-sectional views are shown on the right. A yellow dashed line has been added to the 3D views to indicate more clearly the location of the corresponding 2D transversal sections shown in the panels on the right. Since VM stress distributions are plotted both in the CA healthy wall and fibrous plaque regions, in those cases in which peak stresses are localized in the wall they are colored in gray, while the highest stress regions in the fibrous subset are always displayed in red (see color scales). Red spheres/arrows are used to mark the VM_99_ point location.

Secondly, the VM stress distributions within the CA healthy wall and fibrous plaque subset were analyzed. The corresponding contour plots are shown in Fig. 5 for three example cases, without including the lipid and calcific subsets, as done in Fig. 4. We selected three cases that we considered representative of plaques with a significant calcific or lipidic component or with a more heterogeneous composition (i.e. patients 3, 9 and 5, respectively). The corresponding 3D models of the atherosclerotic CA are provided in the figure as well, together with the transversal slice in which VM_99_ was recorded in the fibrous plaque component. In these plots, the VM_99_ point location is highlighted with a red sphere or arrow. The cross-sectional view of the 3D model is included in Fig. 5 too, in order to provide a clearer visualization of each plaque component morphology and position in the vessel.

The stress distributions represented by the contour plots of Fig. 5 allow us to localize those elements undergoing the highest stresses, where a possible rupture could occur. However, it should be considered that such distributions include both the fibrous plaque subset and the CA healthy wall, thus high VM stress regions could appear both in the plaque and in non-pathological areas. For this reason, in the latter case, the highest stresses in the healthy wall were colored in gray, so as to highlight peak values in the fibrous plaque only, which were displayed in red in all cases.

**Figure 6:**
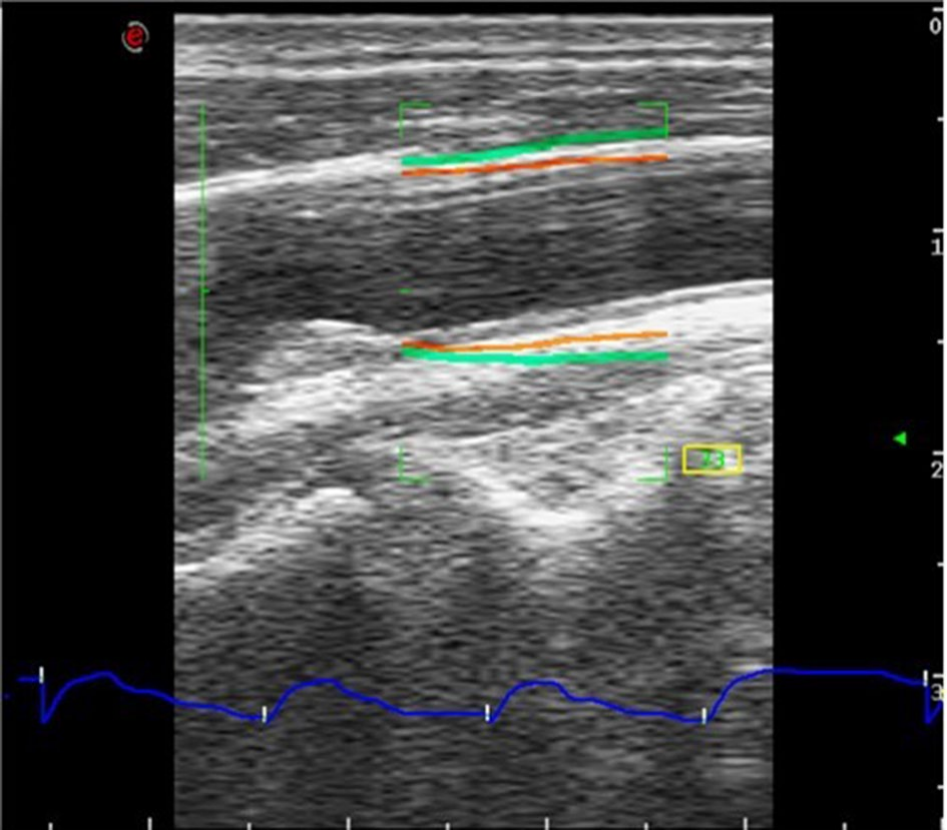
The B-mode image of the CCA on which the vessel distension waveform (in blue), computed using Esaote’s QAS modality, is shown. The QAS mode automatically detects the vessel wall average diameter tracking (in orange) and its consequent amplified movement (in green) associated to wall distension.

Moreover, in Table 2, further data regarding the VM_99_ stress location within the fibrous component are provided for a more complete evaluation. It is known that plaques with a significant lipid component, in which the highest stresses are located within the FC surrounding them, could have a higher risk of rupture, particularly if the FC is thin^36^. This is, for example, the case of patients 5 (Fig. 5), 6, 8, and 9 (Fig. 5), where the VM_99_ stress value is located in the FC at the interface between the lipid pool and the lumen. Thus, in these cases, the thickness of the FC (i.e. of the interface between the lipid component and lumen on the cross-sectional plane where VM_99_ was detected) was measured too (Table 2). On the other hand, when the plaque has a very small or no lipid pool (e.g., patients 1, 2, 3), the highest stresses may appear in the calcific plaque shoulder (see e.g., patient 3 in Fig. 5). Finally, in those cases where the fibrous plaque was also present at the CA bifurcation, the highest stresses were localized in such region (e.g., patients 4, 7, 10). Indeed, a complex geometry such as the carotid bifurcation shows localized extremely high stresses when no pre-stresses are modelled^37^.

## Discussion

This paper proposes a new methodology that allows to reconstruct of an atherosclerotic carotid wall and three different plaque components from CTA images to perform a computational analysis of stress distribution. In order to obtain a wall model that includes the three main components of an atheromatous plaque, the calcific and lipid plaque components were segmented from in vivo CTA images, whereas the fibrous component and the surrounding healthy wall were reconstructed by means of an ad-hoc developed procedure. The advantage brought about by the method proposed here is that the generated models are realistic representations of the atherosclerotic carotid wall, including plaque geometries that are patient-specific, as opposed to other works in which an idealized plaque geometry was used^23,38^. Furthermore, wall reconstruction is derived from 3D images instead of 2D images^12,39^, thanks to which we generated volumetric models and performed a full 3D FEA.

We introduce a procedure to model three different plaque components whose geometries are derived either from CTA images segmentation (for the lipid and calcific ones) or from a new semi-automatic CAD reconstruction method (for the fibrous one, as well as the CA walls surrounding them).

To the best of our knowledge, this work presents for the first time a method to model the patient-specific fibrous content of the plaque, which is not based on the manual segmentation of such component in CTA images. Indeed, it allows to automatically generate models of the plaque fibrous tissues, which could be particularly useful in those cases where difficulties may arise in manually segmenting such component, which is known to be often not easily identifiable in clinical CTA scans^18^. Furthermore, the output of our method allowed us to distinguish the fibrous component of the plaque compared to the healthy vessel, while in other studies, involving both CTA and MR images, this aspect was simplified using the same material properties for both^11,21,22,39,40^, even when the fibrous tissue was detected and segmented. However, since the plaque composition plays a crucial role in determining the stress configuration, this suggests that modelling the plaque as an ensemble of different components with different material properties could provide more accurate results. Adding the fibrous layer around the lipid or/and calcific plaque components in our models yielded a more realistic atherosclerotic CA wall geometry, since actually a FC is always observable at any stage of the plaque progression^4^. To do so in an automatic way, we decided to fill the stenotic regions of the carotid lumen with a fibrous region, representing the presence of an endothelium layer and intimal smooth muscle cells around the other segmented parts of the plaque. On one hand, the geometric model of the fibrous component of the plaque represents that region which could be more likely prone to thickness loss, degradation, and subsequent rupture. On the other hand, FEA allows to analyze and localize the highest stress distribution within it.

The fibrous plaque CAD model we proposed was compared with the manual segmentation of the fibrous plaque tissues provided by the vascular surgeon. Both models (proposed and reference) showed similar stress distributions and locations of the highest stress concentration (see Figs. 3 and 4), in spite of partial geometrical/volume differences between them (see Table 1 and Fig. 4).

Indeed, our method aims at providing models of the fibrous components; as such, these are simplified approximations of the actual plaque geometries, which could suffer from errors in some cases. Even the reference models based on the manual segmentation of the surgeon could suffer from possible inaccuracies, e.g. in those cases in which the fibrous component is hardly detectable in clinical CTA images. However, it should be pointed out that the main target of our work was to develop models able to replicate in a sufficiently accurate way the patient-specific plaque geometry, so that simulation results could give useful indications on stress distributions within the plaque and peaks location, as demonstrated by the obtained results in Fig. 4.

After simulations were performed for all patients, we analyzed the obtained stress distributions within the fibrous plaque subset for each case, for a quantitative evaluation. VM stresses were considered in these analyses, since they are suitable to describe the distribution of loads among different subsets of the simulation, and were also used in several other works in the literature^11,13,15,23,39–41^. The VM_99_ stress values obtained in each case were also reported in Table 2, in order to provide a further parameter that may be useful in assessing the plaque vulnerability or for future patients’ risk stratification. Indeed, we think that localizing those elements undergoing the highest stresses could provide a more complete evaluation of possible rupture sites. The obtained results show that, in plaques with a prevalence of calcific regions, the VM_99_ stresses are mainly localized in the calcific plaque shoulders, while in those with a prevalence of lipid components VM_99_ stresses are within the FC. In these last cases, we also measured the thickness of the FC, which may represent an indicator of plaque vulnerability. When the FC thickness decreased, the VM_99_ stress values increased, confirming that a small FC thickness can increase the probability of having VM stress peaks in such region. Finally, in plaques with a prevalence of fibrous component, the location of VM_99_ stresses was at the CA bifurcation, due to the absence of residual stresses modelling, as previously explained.

The analyses were performed on 10 patients in order to replicate the proposed method on plaques with different geometries and composition. Overall, the results of our simulations can provide valuable indications for plaque vulnerability assessment, particularly if supported in the future by histologic analyses results. At the moment, however, no statistical conclusions on patient risk stratification can be drawn, since the number of cases here considered is very limited.

It is also worth pointing out that the novel developed procedure uses clinical data from different imaging techniques: CTA images were employed for the vessel model creation, while an US echo-tracking modality (Quality Arterial Stiffness (QAS), Esaote, Genova, Italy) was used to set realistic load conditions in simulations. Indeed, the QAS measures of local blood pressure were used as pressure loads applied to the carotid lumen surface, adding a further degree of patient-specificity to the models. The patient-specific pressure data influenced simulation results and should be considered for the assessment of plaque vulnerability.

Our work has some limitations, which will be the object of future developments for further improvements. First, the method we developed is not completely automatized. The main drawback of patient-specific geometries segmentation is that the lipid plaque component is obtained through manual segmentation of images, and often the lumen and plaque geometries are very irregular due to the anatomy and pathology of the carotid vessel. Therefore, some models could be complex and with a time-consuming reconstruction. Secondly, our structural analyses are based on simplifying hypotheses regarding material properties and loads. Even if it is well known that arterial soft tissues are non-linear hyperelastic materials, we assumed elastic and linear material properties according to other works in the literature^15,23,39,40,42–47^. The present study employed material property simplifications to reduce the number of constitutive parameters required for modelling CA atherosclerotic walls. Moreover, due to the lack of clear indication regarding the material properties of atherosclerotic plaques and vessel walls, the simplest model was adopted to provide a practical workflow for the assessment of atherosclerotic carotid plaques based on CTA images^15,23,36,39,40,42–45,47^. Furthermore, our simulations did not account for pre-stresses. The CTA images used to reconstruct the atherosclerotic CA wall geometries provided the lumen configuration in a certain instant of the cardiac cycle, which is characterized by specific load conditions (i.e. blood pressure, residual stresses and axial pre-stretch). Since we lacked such information, this aspect was not considered in our models and simulations, similarly to other works in the literature^13,22,23^. We are aware that these simplifying hypotheses can affect the stress stimulation results. Nevertheless, it should be considered that here the target of FE simulations was mainly to compare the results from different plaque typologies in the same conditions and to assess whether the proposed models worked properly, pointing out those stress distribution differences e.g., between calcific plaques and plaques with a lipid core, that could play an important role in determining their vulnerability.

Finally, one of the major limitations of the study is not considering blood flow: indeed, we know that including the fluid domain and applying a time-dependent pressure waveform to the structural model would lead to a better characterization of hemodynamic loads and to more accurate results in terms of structural stresses. However, we believe that any potential inaccuracy arising from this hypothesis would not have a significant impact on the conclusions drawn from our results, which were mainly focused on evaluating the proposed reconstruction methodology on different patient-specific models of atheromatous CA, starting from the simplest model with the same approximations in all cases.

However, we plan to overcome these limitations in future works, where we could consider the use of nonlinear laws to model healthy vessel and plaque components, for a more accurate assessment of the wall mechanics. Furthermore, we could investigate alternative measures of stress that can significantly differentiate patients with different types of plaque, possibly yielding to an indicator of plaque vulnerability. Additionally, we plan to expand our work by involving a greater number of patients and performing fluid–structure interaction (FSI) analyses (also considering the local pressure waveforms provided by QAS), which could provide important insights into the factors that contribute to plaque rupture.

## Methods

### Patients’ recruitment and image acquisition 

This study was approved by the IRCCS Ospedale San Raffaele ethical committee (record number 110/int/2019, 20 June 2019). All patients enrolled in the study at IRCCS Policlinico San Donato gave written informed consent. All methods were carried out in accordance with relevant guidelines and regulations. Ten patients were scanned using CTA before undergoing carotid endarterectomy (CEA) for asymptomatic stenosis.

Furthermore, patients underwent US image acquisition using a MyLab Eight scanner and a linear probe L4–15 (both from Esaote S.p.A., Genova, Italy). The device was equipped with the QAS analysis software, which implemented a radiofrequency (RF) echo-based arterial wall tracking procedure^48^. In this case, the ultrasound probe was properly positioned to scan the CA longitudinally. The QAS measurements were performed on the far wall in the common carotid artery, with the bifurcation on the left side far from the origin of the bulb. The commercial software tracks arterial wall movements by RF signals during six cardiac cycles in B-mode acquisition, obtaining the distension waveform, which represents the variation in common carotid artery (CCA) diameter as a function of time, by summing the displacement curves of the anterior and posterior walls derived from the processing of RF echo signals (blue curve in Fig. 6). Furthermore, Esaote’s proprietary algorithm computes pressure waveforms from the carotid wall distension waveform and the patient’s brachial pressure. Finally, the commercial software provides the local CA diastolic and systolic pressure (listed in Table 3 as P_D_ and P_S_, respectively) by measured arterial pressure waveform combined with the patient’s brachial pressure values^49^﻿.

**Table 3:** Patients’ local pressure data.

Patient	P_S_ (mmHg)	P_D_ (mmHg)	Local carotid pressure (P_S_–P_D_) (mmHg)
1	161.5	73	88.5
2	158.3	77	81.3
3	112.9	73	39.9
4	148.5	80	68.5
5	151.9	105	46.9
6	159.5	75	84.5
7	105.4	75	30.4
8	116.9	74	42.9
9	135	62	73
10	124.4	93	31.4

P_S_ and P_D_ represent systolic and diastolic local pressure values, respectively.

### Vessel and plaque geometry reconstruction 

**Figure 7:**
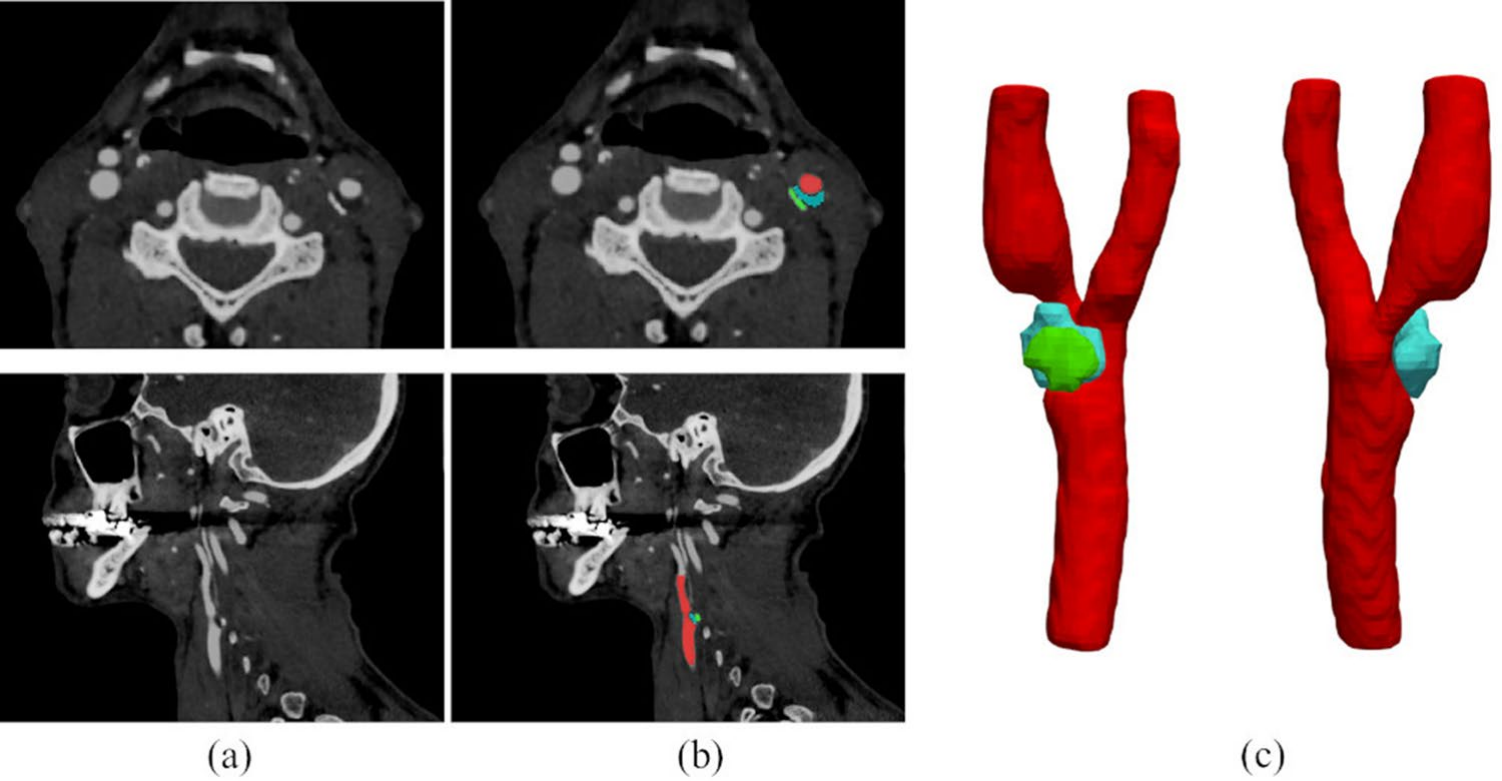
CTA axial and sagittal views for patient 10 (**a**), and corresponding segmentation results (**b**): the lumen is labelled in red, the lipid content of plaque is represented in blue and the calcific content in green. The 3D segmentation results were finally exported in STL format (**c**).

The reconstruction of the atherosclerotic CA wall starts from the segmentation of the DICOM sequences of neck-head CTA scans (Fig. 7a). The segmentation was performed by using a semi-automatic 3D level-set active contour method for the CA lumen and calcific plaque component or manually for the lipid one, using the ITK-Snap v3.6 software^50^. Segmentation is threshold-based, i.e., different thresholds on the Hounsfield units (HU) scale are set by the user to select which region in the image can be considered as a lipidic or calcified plaque or as the CA lumen. Studies generally agree that lipidic and necrotic tissues correspond to “low density” image regions, while fibrotic tissues correspond to “intermediate density” regions and calcium to “high density” ones; however, the reported thresholds are rather variable. In this work, the lumen and the calcific/lipid plaque components were segmented (Fig. 7b) using the HU thresholds listed in Table 4 which are similar on average to those proposed in the literature^17,19,51^.

**Table 4:** Average HU values used for threshold-based segmentation.

Material	HU range
Plaque calcific component	≥ 800
Plaque lipid component	≤ 60
Contrast-enhanced blood	200–700

The accuracy of segmentation was verified by an expert vascular surgeon (D.M.). The surfaces of all the lipid or calcific plaque regions and lumen were exported as STL files (Fig. 7c).

Since the HU intensities of fibrous and muscular tissues of the vessel are similar to those of the surrounding soft tissues, the CA wall and the plaque fibrous component are usually not clearly distinguishable in CTA images. For this reason, in this work such structures were not segmented, but they were reconstructed as CAD models through an ad-hoc procedure developed by using the Rhinoceros v6.13 software (McNeel and Associates, Seattle, WA, USA) integrated with the Grasshopper v1.0 plug-in. The procedure involved the following steps:

i. generation of the vessel wall inner surface by processing the STL surface of the segmented lumen;
ii. generation of the vessel wall outer surface by processing the STL surface of the lumen and plaque components;
iii. generation of a solid geometry by Boolean difference of the inner and outer walls;
iv. filling of the stenotic area of the lumen around the lipid and calcific plaque regions to obtain the fibrous component.

**Figure 8:**
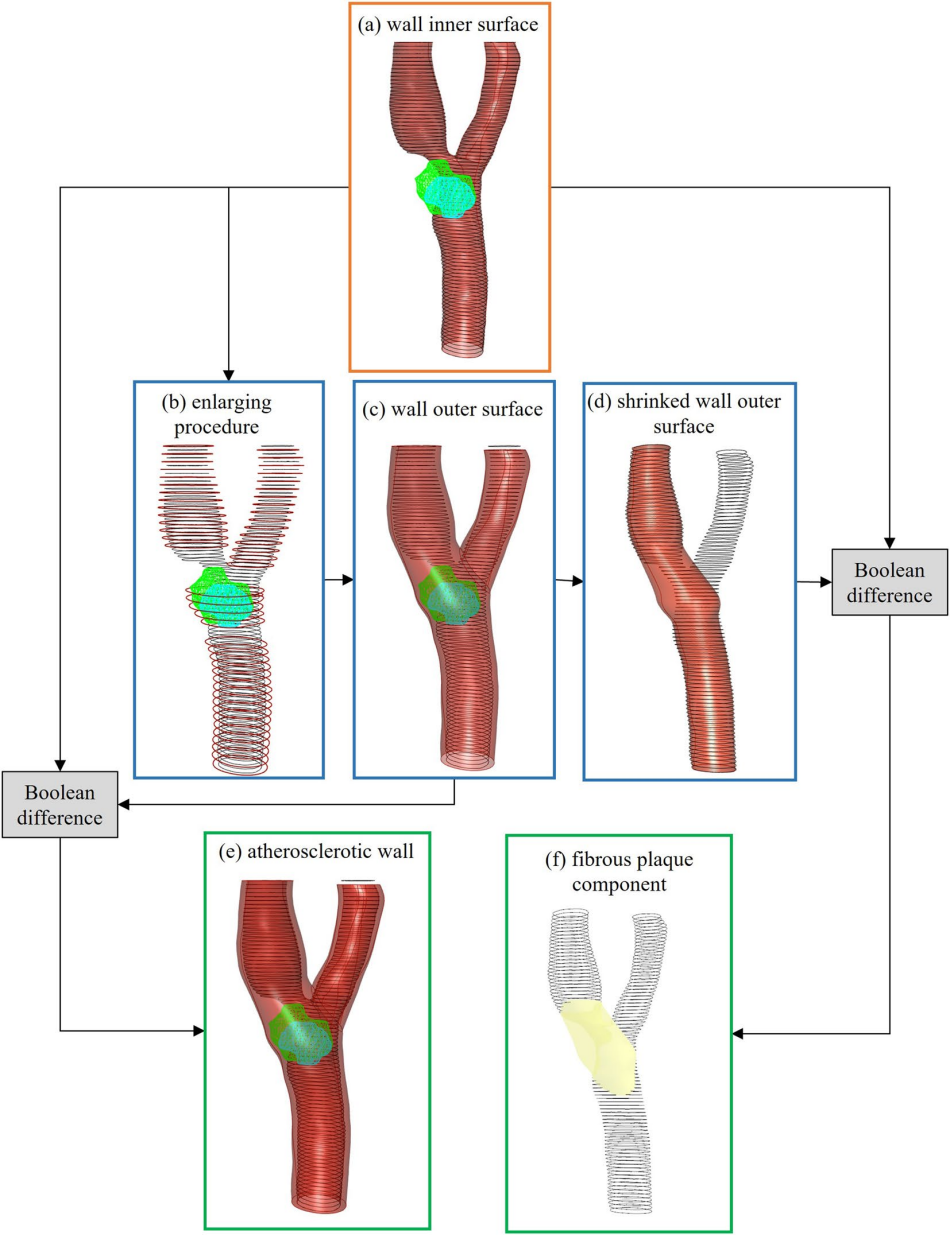
Reconstruction of the atherosclerotic wall solid and fibrous plaque component models. The final atherosclerotic wall geometry (**e**) is obtained as the Boolean difference between the wall inner (**a**) and outer (**c**) surfaces. Panel (**b**) represents the sections enlargement procedure used to include calcific and lipid components of the plaque. To obtain the fibrous plaque component (in yellow in (**f**)), a Boolean difference between the stenotic lumen (**a**) and the shrinked outer wall surface (**d**) is performed. Vessel geometries are shown for patient 10.

In the first two steps, the quickhull algorithm^52^ was used to delimit the region of space enclosing the plaque components with a single surface. The latter, together with the STL lumen, were cut with evenly spaced cross-sectional planes to get the contour curves of the plaques, CCA, internal carotid artery (ICA) and external carotid artery (ECA). In order to obtain a smoother surface, these sections were rebuilt as Non-Uniform Rational B-Splines (NURBS) curves. The lumen surface was then generated by means of the lofting and Boolean operations (Fig. 8a). A fillet was applied at the bifurcation apex to achieve a continuous junction in the CAD model.

In the third step, regarding the outer wall, the lumen and plaque contour curves obtained for each cross-sectional plane were first merged in a single curve using the convex hull algorithm; then, the newly obtained curves were enlarged maintaining the plaque region, defined by the convex hull, enclosed between the inner and outer profile (red curves in Fig. 8b). The enlarging procedure was performed by radially shifting the points that define the luminal profile curves and the plaque sections. Since no information on vessel wall thickness is obtainable from CTA images, the magnitude of radial displacements was defined by assuming that the radius of the outer vessel wall is equal to the distal inner radius of the ICA or ECA increased by 30% of its value^53^. We removed those sections where the outer wall curves, obtained after the enlargement procedure, showed a narrowing (see Fig. 8b), interpolating only the remaining profile curves that model the ideal physiological outer CA wall (Fig. 8c). The solid geometry of the atherosclerotic wall (Fig. 8e) was obtained by Boolean difference between the outer and inner surface.

The final step of the proposed method defines the geometric modelling of the fibrotic component of the plaque within the carotid artery wall (Fig. 8f). We chose to model the fibrous content of the plaque as the region that extends in the stenotic area between the lumen and the outer vessel wall, and that surrounds the other components of the plaque. For this purpose, we applied a Boolean difference between the healthy lumen (Fig. 8d), i.e., the lumen without the region classified as stenotic, and the reconstruction of the pathological lumen (Fig. 8a). The healthy lumen was obtained from the shrinkage of the sections that define the outer wall previously constructed. This step was conducted under the assumption that a healthy carotid lumen should trace the outer wall of the vessel.

### Simulation settings and analysis

**Figure 9:**
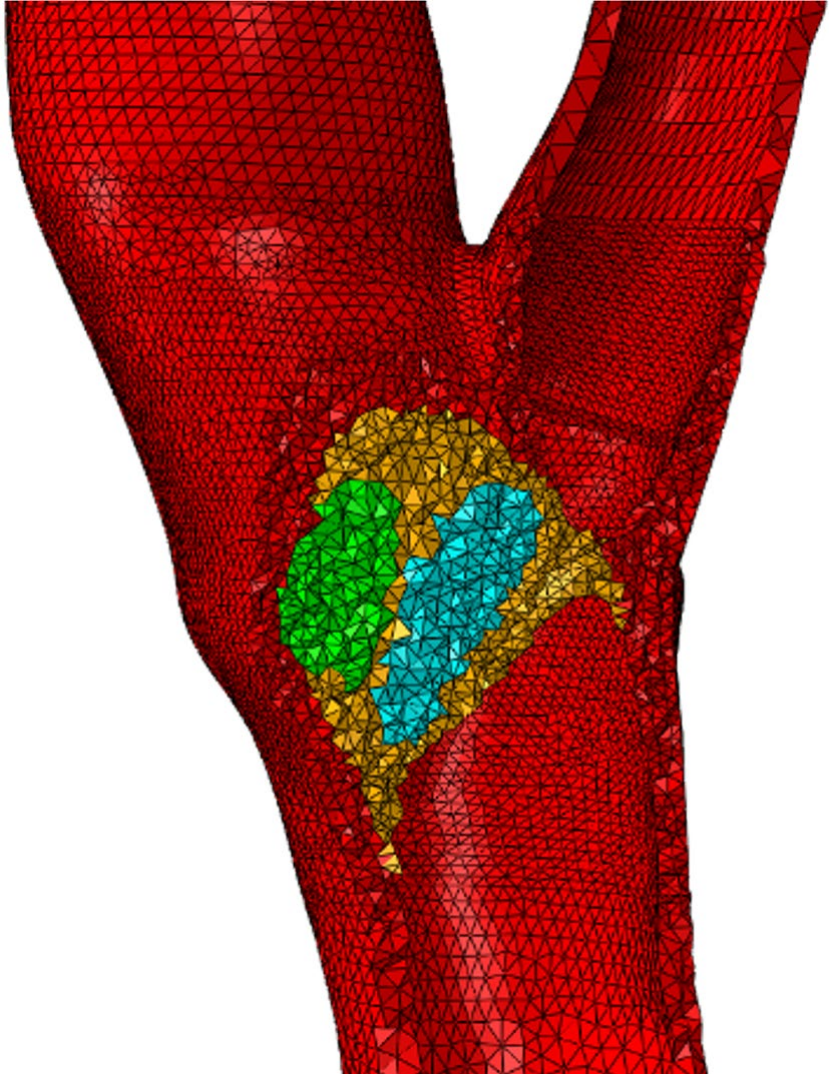
The figure shows a cut-view of the volumetric mesh. To display all different subsets and the corresponding section assignment used for the different components of the wall, the entire mesh was edited removing some tetrahedra. Different colors represent different material properties of components. The subset of elements representing the calcific component is shown in green, the lipid component in blue and the fibrous one in yellow. The remaining elements in red represent the healthy wall.

The reconstructed CA wall geometry was imported into the commercial finite element package Abaqus CAE v6.20 (Simulia, Providence, RI, USA). Static analyses were performed. As shown in Fig. 9, the solid domain was discretized with 4-node tetrahedral elements. The global mesh size was set to 0.8 mm with a local refinement in the plaque region. According to a preliminary mesh convergence analysis, described in the Supplementary Material (Section 1), we chose a local mesh size of 0.3 mm. The partition covers the plaque location: a coarse mesh is used outside the plaque region, whereas a fine mesh is applied inside it.

The materials for both the vessel wall and plaque components were assumed to be nearly incompressible, isotropic, linear elastic, with a 0.49 Poisson’s ratio; instead, a different Young’s Modulus was considered for each of them. The used material properties are reported in Table 5, and were obtained as an average of values available in the literature (considering both computational and experimental studies)^15,23,39,40,42–47^. In order to assign different materials to the elements of the mesh, an identification of different element subsets corresponding to each component of the plaque and CA healthy wall was carried out using an in-house developed Matlab (v2021b, The MathWorks, Natick, MA, USA) algorithm. Elements of the tetrahedral mesh that were enclosed within the STL surface of each plaque component were selected as follows: the minimum distance vector between each triangle of the STL plaque surfaces and each tetrahedron of the mesh was computed and then compared with the normal to the triangle face; the tetrahedron was selected as element of a subset if the minimum distance vector from the triangle had the same direction of its normal. The subsets and the corresponding material section assignment used for the different components of the wall are shown in Fig. 9.

**Table 5:** Material properties set in FEA.

Material	Young’s modulus (kPa)
Plaque calcific component	20,000
Plaque lipid component	4
Plaque fibrous component	400
CA healthy wall	550

The load conditions of the CA wall were defined as follows, also based on previous works in the literature: the distal ends of the ICA and ECA and the proximal end of the CCA were constrained in the longitudinal and circumferential directions; a constant uniform pressure load was then applied to the lumen surface, as reported in^13,22^. Thanks to the availability of clinical data derived from the US QAS mode, we could use patient-specific pressure values equivalent to the differential local blood pressure, i.e., the difference between systolic and diastolic local pressure. The differential local pressure values, used as the magnitude of the uniform pressure load employed in simulations, are listed in Table 3.

### Post-processing of simulation results 

A total of 10 simulations were performed and the VM stress values were analyzed. We supposed that the fibrous plaque component, surrounding the other components and interfacing with the lumen surface, could represent the more critical region in which rupture is likely to occur for vulnerable plaques. Thus, for each element of the fibrous subsets, the tetrahedral volume, together with the VM stress and coordinates of the centroid corresponding to the tetrahedron, were extracted. We derived the VM value corresponding to the 99% of the cumulative volume of the elements of the fibrous plaque and denoted it as VM_99_. The latter was obtained as follows: first, all elements of the subset were sorted based on increasing stress values, and the cumulative volume of the sorted elements was calculated, until it reached 99%; the element associated to this cumulative volume was designated as the element in which the VM_99_ stress occurred. This means that the 99% of the fibrous component volume shows VM stress values which are lower or equal to VM_99_.

Besides, to assess our geometric reconstruction method, we first compared the fibrous plaque CAD model, automatically generated using the proposed approach, with the one obtained from the manual segmentation of CTA images performed by the vascular surgeon. The surgeon provided the manual segmentation based on 2D contouring of the plaque fibrous component for all the 10 patients.

Model-evaluation included the following three different analyses.

The obtained fibrous plaque 3D models were initially evaluated through a mutual distance analysis, which aimed at investigating differences between the proposed reconstructed geometry and the manually segmented geometry of the plaque fibrous components.

Secondly, three patients (i.e. patients 1, 4 and 7) were selected, and for them two sets of simulations were performed: in the former, the fibrous plaque CAD model was used together with those of the healthy CA wall and calcific/lipid plaque components (generated as described before); in the latter, the fibrous plaque CAD model was replaced with the one derived from manual segmentation, while the CA wall and calcific/lipid plaque component models remained the same. We refer to these two models as the “proposed model” and “reference model”, respectively. The results of the two simulations were then quantitatively compared. Patients 1, 4 and 7 were selected for this analysis as, also based on the surgeon’s opinion, they seemed representative of different plaque typologies, i.e.: a calcific plaque, with no lipid component (patient 1); a plaque with both a lipid and calcific component, but with a prevalence of this latter, and a significant fibrous tissue percentage (patient 4); a plaque with both a lipid and calcific component, but with a prevalence of the former, and again a significant fibrous tissue percentage (patient 7).

The third step involved a sensitivity analysis, in which the Young’s modulus assigned to the fibrous component of patients 1, 4 and 7 was varied by ± 10%, ± 20%, ± 50% and stress distributions were evaluated in each case, comparing results obtained using either the reference or proposed model in simulations. Indeed, in this work, we initially considered an elastic modulus of 400 kPa for the fibrous plaque and of 550 kPa for the carotid wall, which are quite similar. This analysis aims at demonstrating that the differences obtained in terms of stresses between the reference and proposed models are small even when the elastic modulus of the fibrous plaque varies.

### Supplementary Information

Supplementary Information.

## Data Availability

Data analyzed during the current study are available from the corresponding author on reasonable request.
