# Supplementary material for: 3D patient-specific modeling and structural finite element analysis of atherosclerotic carotid artery based on computed tomography angiography
Source: Sci Rep. 2023 Nov 14;13:19911. doi: 10.1038/s41598-023-46949-5 (PMC10645924; doi:10.1038/s41598-023-46949-5)
Supplement: Supplementary file 1 — Supplementary Information. [file 41598_2023_46949_MOESM1_ESM.pdf]

Supplementary Material

3D Patient-specific Modeling and Structural Finite Element Analysis of Atherosclerotic Carotid Artery Based on Computed Tomography Angiography

Nicoletta Curcio, Antonio Rosato, Daniela Mazzaccaro, Giovanni Nano, Michele Conti, Giulia Matrone*

*** Correspondence:** Giulia Matrone: giulia.matrone@unipv.it

1 Mesh Convergence Analysis

The mesh density has an impact on the element subsets selection corresponding to the different plaque components and CA healthy wall and consequently on the simulation results. Therefore, a preliminary mesh-convergence analysis was performed for one patient. Different meshes were created on the same CA model: a partition was used to delimit the full plaque region and the local seed size was varied to assess the difference on the estimated plaque volume from STL files and on the VM_{99} stress results. The local seed size was decreased with 0.1 mm step going from 0.8 mm to 0.2 mm. Plaque volume and VM_{99} were investigated. More specifically, concerning the plaque volume, the difference between the volume enclosed into the plaque STL files derived from CTA images (i.e., reference volume given by the sum of lipid and calcific volumes, which is 113.151 mm^3) and the volume predicted by the lipid and calcific plaque subsets was assessed (Δvolume). For what concerns the VM_{99} stress instead, the difference between the results obtained using the coarser mesh and the actual mesh density was assessed (ΔVM_{99}).
The final size of the seeds was chosen when the variation of these parameters for two consecutive mesh sizes was considered negligible. Data reported in Table 1S show that the mesh refining with local seed size of 0.4 mm and 0.3 mm produced a negligible difference in the computed VM_{99}, creating a plateau in the simulation results.
Thus, since further mesh refining would dramatically increase the computational requirements without significantly improving the plaque volume estimation, we chose a final mesh size of 0.3 mm inside the plaque region (including the fibrous, lipid and calcific components). The global mesh size (outside the plaque) was instead set to 0.8 mm.

Table 1S. Mesh convergence analysis

Local seed size (mm)	Number of elements	Plaque volume (mm^3)	Δvolume (mm^3)	VM_{99} stress (kPa)	ΔVM_{99} stress (kPa)
0.8	24007	112.9137	0.2373	16.3847	-
0.7	32166	113.0429	0.1081	17.4492	-1.0645
0.6	47761	112.937	0.214	17.7448	-1.3601
0.5	62723	112.9719	0.1791	18.4456	-2.0609
0.4	107947	112.6837	**0.4673**	19.2946	**-2.9099**
0.3	235362	112.6936	**0.4574**	19.1353	**-2.7506**
0.2	580821	112.4744	0.6766	21.0986	-4.7139

Values highlighted in bold have minimal variation.

2 Geometric Comparison of Fibrous Components Models

3D images obtained from the mutual distance analysis between the proposed reconstructed geometry and the manually segmented geometry of the plaque fibrous components, presented in the paper for patients 1-4-7, are here provided also for the seven remaining patients in Fig. 1S. Quantitative results for all 10 patients are reported in Table 2S.

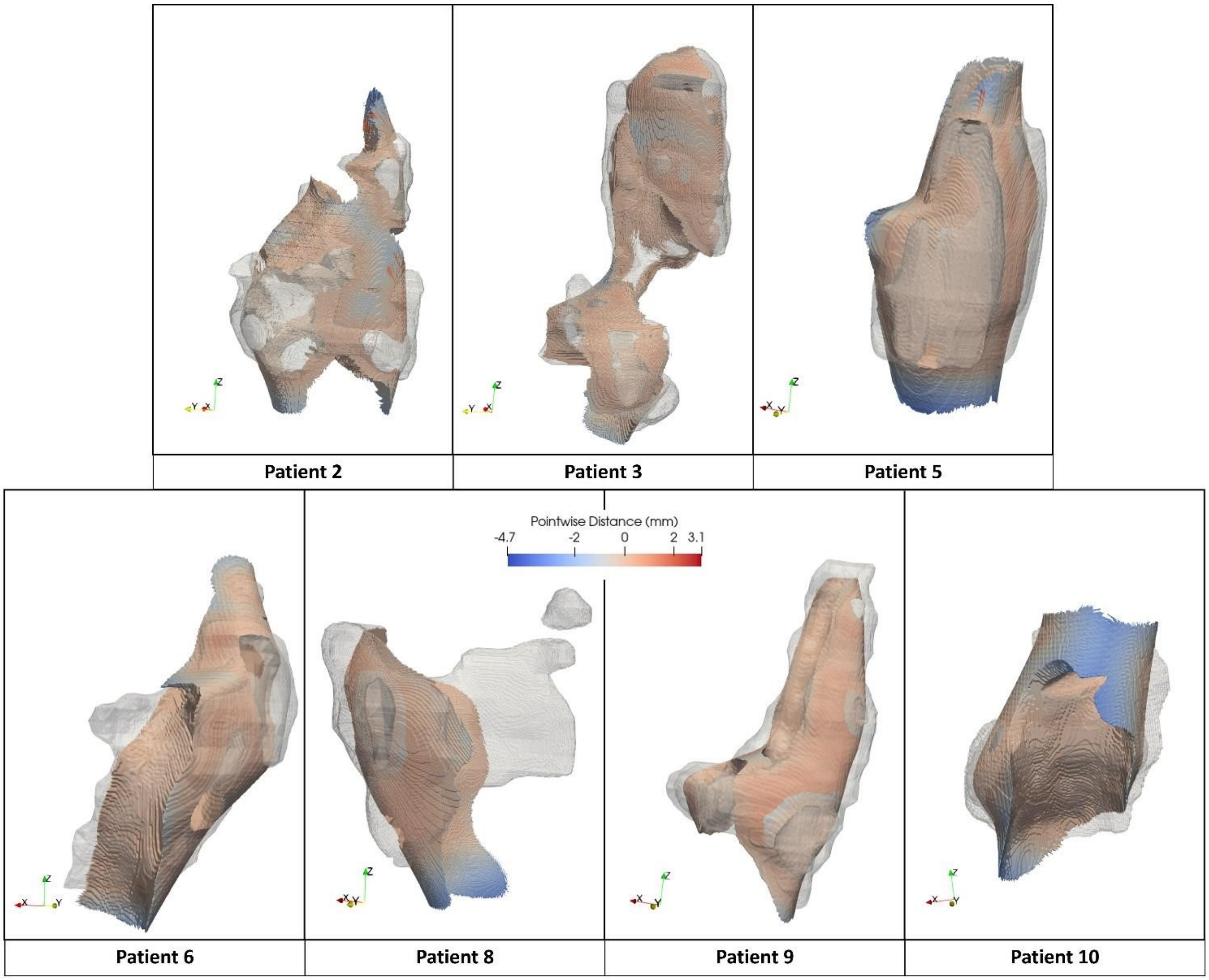

Fig. 1S. Pointwise distances between the reference (in transparency) and proposed models of the fibrous plaque component for patients 2, 3, 5, 6, 8, 9 and 10. The same color scale of Figure 2 has been applied.

Table 2S. Mutual distances and volume differences between the reference and proposed fibrous plaque models.

Patient	Distance* (mm)	Volume difference (%)
1	-1.356±1.079	78.79
2	-0.171±0.429	-23.51
3	-0.015±0.257	-37.95
4	-0.509±0.746	12.67
5	-0.183±0.587	-31.81
6	-0.067±0.352	-38.25
7	-0.399±0.786	21.83
8	0.017±0.644	-67.47
9	0.069±0.283	-49.92
10	-0.256±0.677	-24.75

*Distances are provided as mean ± standard deviation.

It should be noted that percentage volume differences provided in Table 2S have been computed using volumes derived from geometrical models of the plaque, while volumes reported in Tables 1 and 2 in the paper have been computed on the FE models in Abaqus. For this reason, some slight differences can be observed e.g. between volume percentage differences of patients 1-4-7 in Table 2S and Table 1. Nevertheless, the plot in Fig. 2S demonstrates that volumes derived from the geometrical and FE model of the same plaque are very similar.

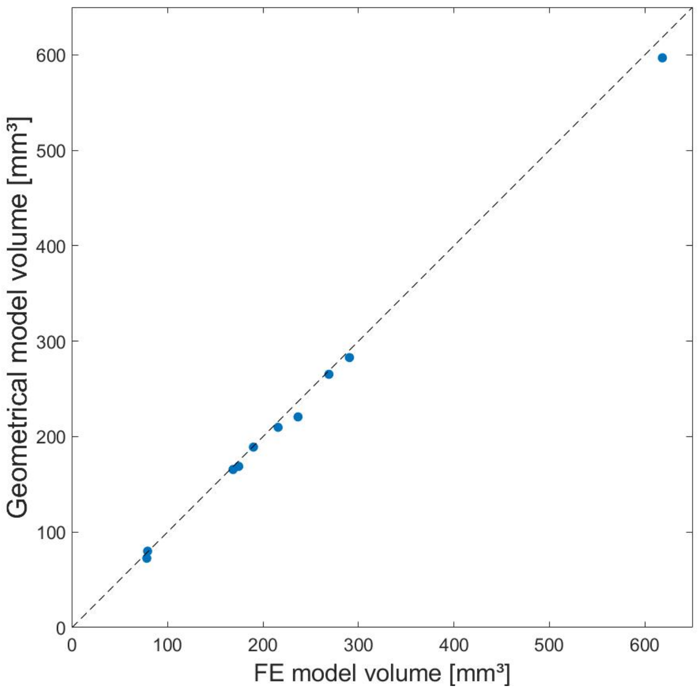

Fig. 2S. Comparison of volumes computed from the geometrical and FE model of the fibrous plaque for each patient (blue dots). In this case, the two volume computation approaches have been compared considering the "proposed model" of the fibrous plaque, i.e. the one obtained with our reconstruction method.

3 Sensitivity Analysis of Stress Distributions to Variations of the Fibrous Component Young's Modulus

Since the Young's moduli set for the plaque fibrous component and carotid healthy wall were quite similar (i.e. 400 kPa vs. 550 kPa), a further analysis was carried out to evaluate if more significant differences could appear between the stress distributions obtained using the reference and proposed models when the Young's modulus of the fibrous component varies. The considered variations were ±10%, ±20%, ±50% of the Young's modulus initially employed (i.e. 400 kPa). Analyses were carried out for patients 1-4-7.

In Fig. 3S, the trend of VM_{99} values vs. volume is compared for all the analyzed cases in the 3 selected patients. The plots demonstrate that the same differences/similarities observed between the reference and proposed model curves for the initial Young's modulus value (400 kPa) in Figure 3 are still present even when this parameter varies.

Tables 3S-4S-5S also provide VM_{99} values quantitatively in all the analyzed cases. Indeed, the VM_{99} percentage difference between the reference and proposed models for each patient doesn't show significant variations when the Young's modulus varies within the considered range.

Table 3S. VM_{99} for patient 1 using different Young's moduli for the fibrous tissues

Fibrous plaque Young's modulus variation	Proposed model VM_{99} (kPa)	Reference model VM_{99} (kPa)	VM_{99} percentage difference (%)
-50% (200 kPa)	28.95	52.47	-44.83
-20% (320 kPa)	38.22	59.08	-35.32
-10% (360 kPa)	40.79	62.38	-34.61
0% (400 kPa)	43.23	65.71	-34.22
+10% (440 kPa)	45.52	69.23	-34.25
+20% (480 kPa)	47.71	72.59	-34.27
+50% (600 kPa)	53.77	81.68	-34.18

Table 4S. VM_{99} for patient 4 using different Young's moduli for the fibrous tissues

Fibrous plaque Young's modulus variation	Proposed model VM_{99} (kPa)	Reference model VM_{99} (kPa)	VM_{99} percentage difference (%)
-50% (200 kPa)	31.33	30.17	3.85
-20% (320 kPa)	35.55	37.23	-4.54
-10% (360 kPa)	36.69	39.31	-6.67
0% (400 kPa)	41.14	37.86	8.66
+10% (440 kPa)	38.96	42.97	-9.33
+20% (480 kPa)	40.12	44.54	-9.92
+50% (600 kPa)	43.43	49.42	-12.13

Table 5S. VM_{99} for patient 7 using different Young's moduli for the fibrous tissues

Fibrous plaque Young's modulus variation	Proposed model VM_{99} (kPa)	Reference model VM_{99} (kPa)	VM_{99} percentage difference (%)
-50% (200 kPa)	13.84	15.22	-9.02
-20% (320 kPa)	16.91	18.64	-9.25
-10% (360 kPa)	17.73	19.68	-9.89
0% (400 kPa)	18.48	20.69	-10.69
+10% (440 kPa)	19.18	21.66	-11.47
+20% (480 kPa)	19.82	22.62	-12.36
+50% (600 kPa)	21.54	25.30	-14.88

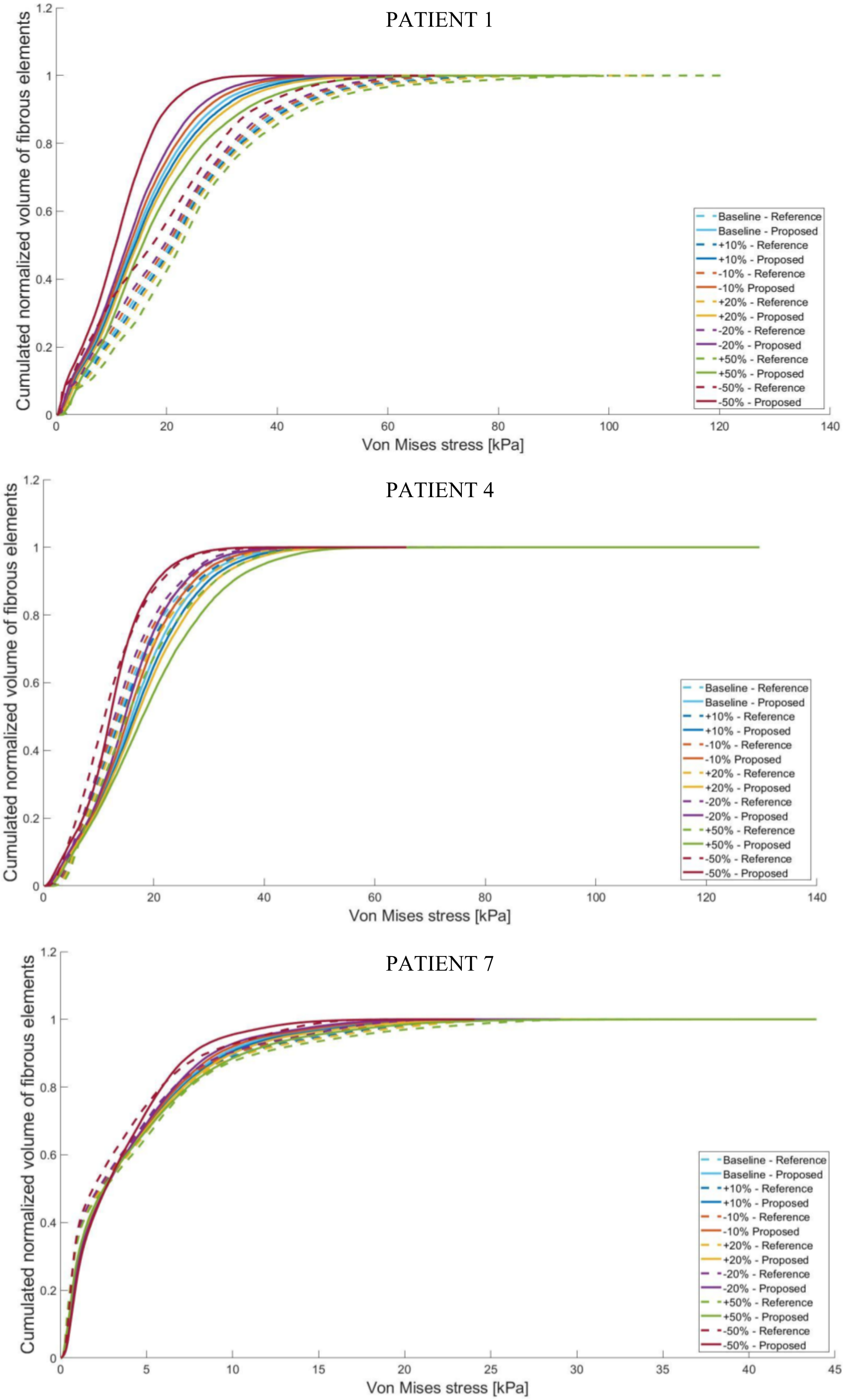

Fig. 3S. Trend of Von Mises stress vs. cumulated volume of the fibrous elements for patients 1, 4 and 7. Different colors have been used for each variation of the fibrous tissue Young's modulus (±10%, ±20%, ±50%), with "Baseline" referring to the initial value (400 kPa). Dashed and solid curves refer to results obtained with the reference and proposed models, respectively.

Finally, Fig. 4S compares the 2D stress distributions in the slice where the VM_{99} is detected. The distributions are shown for the -50% case only, as an example, since it represents the case with the greatest difference between the Young's moduli of the fibrous tissue and wall (i.e. 200 kPa vs. 550 kPa) among the values considered. These plots also confirm that stress distributions for the reference and proposed models for each patient remain similar even when the Young's modulus of the fibrous plaque is 200 kPa, as observed in the initial case in Figure 4.

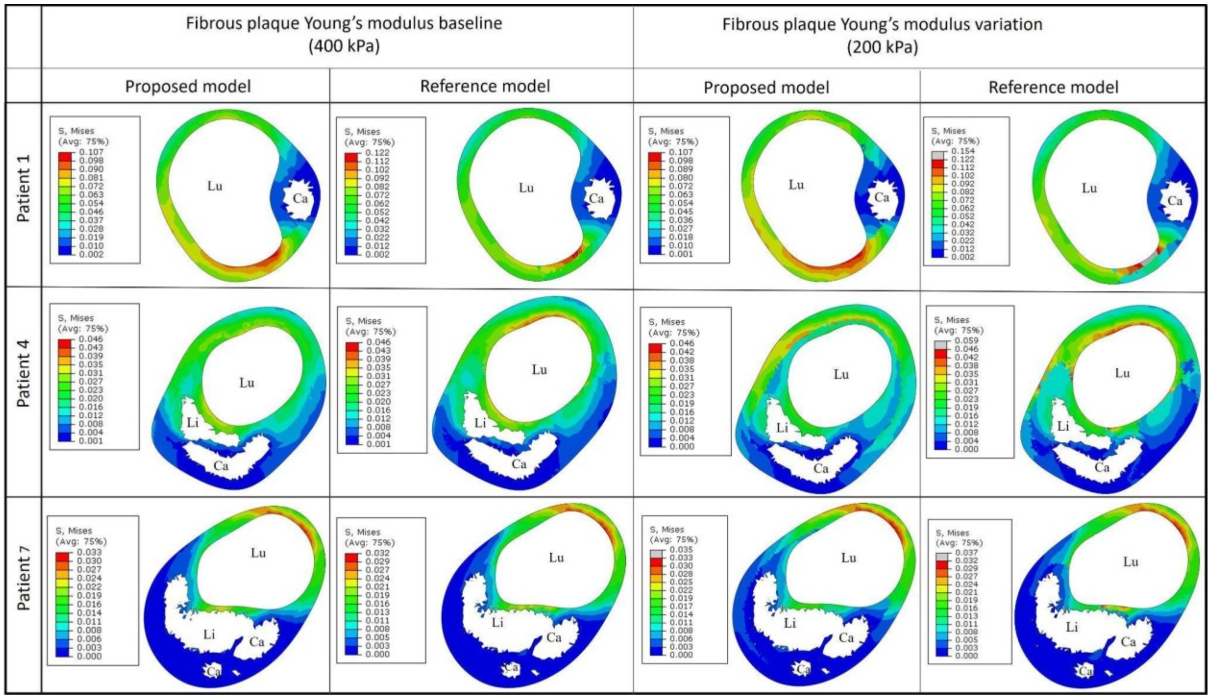

Fig. 4S. 2D stress distributions obtained with the proposed and reference models for patients 1, 4 and 7 when employing different Young's moduli values (400 kPa and 200 kPa) for the fibrous plaque component.
